# Anti-Jamming Communication Using Imitation Learning

**DOI:** 10.3390/e25111547

**Published:** 2023-11-16

**Authors:** Zhanyang Zhou, Yingtao Niu, Boyu Wan, Wenhao Zhou

**Affiliations:** 1 Sixty-Third Research Institute, National University of Defense Technology, Nanjing 210007, China; zzy285518423@hotmail.com (Z.Z.); 18168761685@163.com (W.Z.); 2 Fundamentals Department, Air Force Engineering University of PLA, Xi’an 710051, China; wby1196874871@163.com

**Keywords:** anti-jamming communication, spectrum decision, imitation learning, expert strategy

## Abstract

The communication reliability of wireless communication systems is threatened by malicious jammers. Aiming at the problem of reliable communication under malicious jamming, a large number of schemes have been proposed to mitigate the effects of malicious jamming by avoiding the blocking interference of jammers. However, the existing anti-jamming schemes, such as fixed strategy, Reinforcement learning (RL), and deep Q network (DQN) have limited use of historical data, and most of them only pay attention to the current state changes and cannot gain experience from historical samples. In view of this, this manuscript proposes anti-jamming communication using imitation learning. Specifically, this manuscript addresses the problem of anti-jamming decisions for wireless communication in scenarios with malicious jamming and proposes an algorithm that consists of three steps: First, the heuristic-based Expert Trajectory Generation Algorithm is proposed as the expert strategy, which enables us to obtain the expert trajectory from historical samples. The trajectory mentioned in this algorithm represents the sequence of actions undertaken by the expert in various situations. Then obtaining a user strategy by imitating the expert strategy using an imitation learning neural network. Finally, adopting a functional user strategy for efficient and sequential anti-jamming decisions. Simulation results indicate that the proposed method outperforms the RL-based anti-jamming method and DQN-based anti-jamming method regarding solving continuous-state spectrum anti-jamming problems without causing “curse of dimensionality” and providing greater robustness against channel fading and noise as well as when the jamming pattern changes.

## 1. Introduction

With the large-scale application of autonomous driving [1], Intelligent Vehicles [2], drone light formation show [3], Internet-of-Things [4] and other technologies, information technology is deeply integrated into our lives. As the foundation of information technology, wireless communication is easy to be affected by user interference and malicious jamming due to the openness of its channel, which reduces the efficiency of its actual deployment [5,6,7,8].

Addressing the impact of such issues is crucial in wireless communication systems [9]. Therefore, making timely and effective spectrum decisions for different jamming patterns is practical in the anti-jamming process of wireless communication systems. Researchers have proposed various anti-jamming methods, including the extensively studied intelligent anti-jamming technology based on machine learning. Table 1 shows a summary of related research, and mainly introduces the research status of intelligent anti-jamming technology based on federated learning (FL), meta-learning (Mate-L), reinforcement learning (RL), and deep reinforcement learning (DRL). To restore normal communication quickly and stably when the wireless communication system is faced with jamming, researchers use the method based on automatic control  [10,11] to prevent jamming. To address the issue of local data secrecy during multi-agent anti-jamming communication, researchers employ federated learning (FL) [12,13,14] for anti-jamming purposes. To enable agents to learn how to learn during anti-jamming communication, researchers have utilized meta-learning [15,16,17] for anti-jamming. To facilitate anti-jamming decision-making as the state changes, researchers employ reinforcement learning (RL)  [18,19,20] for anti-jamming communication. To mitigate the “curse of dimensionality” problem associated with RL, researchers have extensively investigated the use of DQN  [21,22,23] for anti-jamming communication. DQN has shown promise in the field of anti-jamming decision-making due to its ability to make decisions while learning and adapting to new jamming scenarios. Building upon this foundation, scholars worldwide have proposed improved methods such as multi-agent layered Q-learning (MALQL) and UCB-DQN  [23].

The anti-jamming method based on automatic control can quickly and effectively deal with the current jamming and make appropriate anti-jamming actions, but this method cannot accumulate historical experience and cannot effectively predict the state change before anti-jamming. The intelligent anti-jamming communication methods are based on FL sacrifice performance for the sake of data security, and the performance is limited by the communication efficiency between agents. The intelligent anti-jamming communication method based on Meta-L requires high computing power, which is not practical for anti-jamming communication. The intelligent anti-jamming communication method based on RL fails to address the problem of continuous-state space and faces the challenge of the exponential growth of “state-action” pairs, known as the “dimension disaster”. The intelligent anti-jamming communication methods based on DQN have low sampling efficiency for state samples and limited utilization of historical state information, which may result in considerable time required for convergence even in typical or similar jamming scenarios. Furthermore, DQN imposes high requirements on the neural network, and an inappropriate neural network can lead to instability in the intelligent anti-jamming communication system based on DQN.

In our previous work, we mainly studied the intelligent anti-jamming communication technology based on RL  [5,24] and DQN [25], improved some technical details, and combined these with UCB technology. However, in this process, we find that both RL and DQN are faced with the dilemma of exploration and utilization, which requires a lot of time to explore the environment, and the ability to explore the environment in the utilization stage is poor. If the interference style is switched at this time, the anti-jamming ability of the system will be more significantly affected. In practice, jammers often enhance the difficulty of jamming by switching jamming patterns or dynamically adapting parameters. Consequently, efficiently utilizing experience from typical or similar scenarios to make effective anti-jamming decisions has become a challenging problem. With the advancement of research on inverse reinforcement learning, the method of imitation learning  [26,27,28] can be employed to make timely and effective anti-jamming decisions in the face of typical and similar scenarios. Therefore, we can obtain an efficient spectral decision-making method by analyzing historical samples, and take it as an expert strategy(ES), to form an expert trajectory(ET). Using imitation learning neural network(ILNN), we can obtain a user strategy capable of making efficient decisions, to significantly improve the response speed and robustness of decision-making. Unlike reinforcement learning, which needs to maintain a Q-table or DQN so that each state-action has its corresponding Q-value, the imitation learning method proposed in this paper treats the decision problem as a fitting problem of state-to-action selection probability. ILNN fits the action selection probability in the next time slot according to the state of several past moments. The system selects the anti-jamming action according to the selection probability. Therefore, there is no fear of the curse of dimensionality.

Based on this view, this manuscript proposes the following spectrum anti-jamming method using IL for scenarios where varied conventional jamming appears alternatively:

- First, a proposed Expert Trajectory Generation Algorithm (ETGA) serves as the expert strategy. Anti-jamming decision-making is carried out on historical state samples (HSS). To enable the system to stably avoid jamming signals, the expert strategy is generated accordingly.
- Next, Imitation Learning Neural Networks are proposed. It takes historical state samples as input and the expert strategy generated by ETGA as expected output. Through network training, a user strategy (US) can be obtained, enabling it to make decisions according to the state of several previous time slots.
- Finally, the user strategy is used as a function to obtain anti-jamming decision actions efficiently and sequentially based on the states of multiple previous timeslots in the anti-jamming process.

The remainder of this manuscript is organized as follows: Section 2 introduces the system model and problem formulation. Section 3 explains the method. Section 4 presents the simulation results. Finally, Section 5 summarizes the conclusions.

## 2. System Model and Problem Formulation

This section presents the system model and problem formulation of this manuscript. The system comprises a transmitter, receiver, agent, and jammer. The problem formulation introduces the reward function and objective function discussed in this manuscript.

### 2.1. System Model

**Figure 1:**
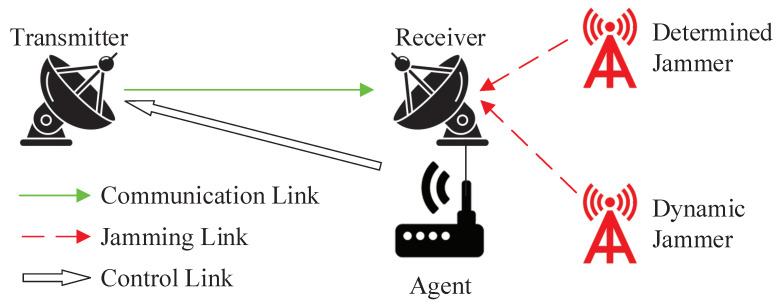
System model.

The system model in this manuscript, depicted in Figure 1, considers a wireless communication scenario where multiple malicious jammers are present. The transmitter *T* sends the signal S(t) while the receiver *R* is being maliciously jammed by both the determined jammer and the dynamic jammer. The agent at the receiver perceives the state s, and learns and decides on the anti-jamming action a, and transmits it to the transmitter through an independent control link to modify the communication action [5].

### 2.2. Problem Formulation

This subsection outlines the reward function and objective function for this problem. The reward function is dependent on the current state and the chosen action. The objective function aims to minimize the disparity between the reward obtained from the user strategy and the reward achieved from the expert strategy.

#### 2.2.1. Reward Function

The communication band is divided into *N* non-overlapping channels, and the state s is represented by a N×1 array where each element corresponds to the strength of the noise plus jamming signal $s_{i}$ of each channel:

(1) $$s={\left[s_{1},s_{2},\cdots ,s_{N}\right]}^{T}.$$

Similarly, the action a is defined as a N∗1 array with only one element being 1 and the others being 0.

(2) $$a={\left[0,0,\cdots ,1,\cdots ,0\right]}^{T}.$$

The reward *r* obtained by selecting action a in state s is given by the following:

(3) $$r=R\left(a,s\right)=\left\{\begin{array}{c} 0,s_{i}⩾\varepsilon \\ 1,s_{i}<\varepsilon \end{array}\right.,a_{i}=1,$$

where ε is the energy threshold for jamming plus noise.

#### 2.2.2. Objective Function

To solve the anti-jamming problem, we use imitation learning to learn from the expert strategy and minimize the difference between the user reward expectation (URE) and the expert reward expectation (ERE) under policy π.

IL aims to learn tasks from expert strategy, which can extract information about actions and the surrounding environment, and learn the relationship between states and actions.

For a given expert strategy $\pi_{E}$, the expert reward expectation can be calculated according to this strategy $\mu_{E}=\mu \left(\pi_{E}\right)$. Generally speaking, expert strategy $\pi_{E}$ is often manifested as expert strategy, that is, the action switching process under the expert strategy $\pi_{E}$. For *m* expert strategies ${\left[a_{0}^{\left(i\right)},a_{1}^{\left(i\right)},a_{2}^{\left(i\right)},\cdots \right]}_{i=1}^{m}$ the expert reward expectation $\mu_{E}$ is calculated as follows:

(4) $$\mu_{E}=\mu \left(\pi_{E}\right)=\frac{1}{m}\sum_{i=1}^{m}\sum_{t=0}^{\infty }R\left(a_{t},s_{t}\right).$$

At the same time, for the user who adopts user strategy π, its user reward expectation (URE) can be expressed as follows:

(5) $$\mu \left(\pi \right)=E\left[\sum_{t=0}^{\infty }R\left(a_{t},s_{t}\right)|\pi \right].$$

We expect the Agent to minimize the difference between the user reward expectation μ(π) and the expert reward expectation $\mu_{E}$ under policy π:

(6) $$\arg\min{∥\mu \left(\pi \right)-\mu_{E}∥}_{2}$$

## 3. Method

This section proposes an anti-jamming method using imitation learning based on the above system model for the system model in Section 2. First, the process structure of the designed anti-jamming method using imitation learning is given, then the Expert Trajectory Generation Algorithm is given, and finally, the design of imitation learning neural network is given.

### 3.1. Process Structure

**Figure 2:**
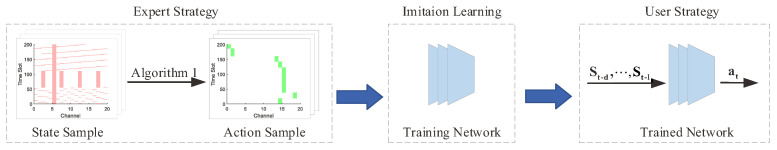
Method process structure.

As illustrated in Figure 2, the process structure of the designed anti-jamming method using imitation learning is proposed for intelligent spectrum anti-jamming in the scenario described in Section 2.

The first step is to obtain the state sample S through the perception of the RF environment. The expert strategy $A_{E}$ is then generated through the expert strategy, as shown in Algorithm 1,

(7) $$A_{E}=F\left(S\right).$$

The expert strategy is generated posteriorly, allowing the algorithm to select the action $a_{t}$ based on either the state change before or after the timeslot, maximizing the total communication reward of this trajectory. The resulting expert strategy $A_{E}$ can be expressed as

(8) $$\begin{array}{cccc} A_{E}=\; & \left[a_{1},a_{2},\cdots ,a_{M}\right]= & F\left(S\right)=\underset{a_{i}}{\arg\max}\sum_{i=1}^{M}R\left(a_{i},s_{i}\right)s_{i}\in & S \end{array},$$

which is an action sequence equal to the length of the training sample S timeslot.

In anti-jamming practice, the network parameters $\theta_{N}$ are adjusted through learning and adjusting to obtain the maximum reward by observing historical jamming information and obtaining the next action through the function $f_{\theta_{N}}\left(•\right)$

(9) $$\begin{array}{c} a_{t}=\underset{\theta_{N}}{\arg\max}R\left(a_{t},s_{t}\right)=\underset{\theta_{N}}{\arg\max}R\left(f_{\theta_{N}}\left(s_{t-d},\cdots ,s_{t-1};a_{t-d},\cdots ,\;a_{t-1}\right),s_{t}\right). \end{array}$$

The ILNN is trained to imitate the expert strategy to obtain the user strategy, and the function ${\hat{f}}_{\theta_{N}}\left(•\right)$ is used as the efficient decision function during the process, obtaining the next action $a_{t+1}$ through the following formula:

(10) $$a_{t+1}={\hat{f}}_{\theta_{N}}\left(s_{t-d+1},\cdots ,s_{t};a_{t-d},\cdots ,\;a_{t-1}\right).$$

Implementing this algorithm faces two challenges, which are addressed in the next section:

- Obtaining the expert strategy through the Expert Trajectory Generation Algorithm;
- Training the Imitation Learning Neural Network based on the expert strategy;

**Algorithm 1** Expert Trajectory Generation Algorithm (ETGA)**Input:** Time-frequency matrix **RF** (N*M).**Output:** Expert selection sequence **ESS** (1*M).**initialize: ESS** = zeros(1,M); Consecutive unjammed channel start timeslot **S1** = 1;Consecutive unjammed channel end timeslot **S2** = TL.**while** min(ESS) == 0 **do**   J = zeros(N,1)   **for** ii = 1:N **do**     **if** sum(RF(ii,S1:S2) > 0) == 0 **then**        J(ii) = S2+1;     **else**        J(ii) = find(RF(ii,S1:S2) > 0,1)+S1−1     **end if**   **end for**   [M,N] = max(J);   ESS(S1:M−1) = N*ones(M−S1,1);   S1 = M;**end while**

### 3.2. Expert Trajectory Generation Algorithm (ETGA)

The Expert Trajectory Generation Algorithm (ETGA) is shown in Algorithm 1. The algorithm selects the trajectory according to the time-frequency state matrix of N∗M based on the expert strategy, ensuring no collision with the jamming channel and that the communication signal stays in the same channel as long as possible for stable and sequential communication.

The expert strategy corresponding to the Expert selection sequence (ESS) in Algorithm 1 is

(11) $$a_{E}\left(n\right)=\left\{a_{\mathrm{Scheme}\left(n\right)}=1\right\}={\left[0,\cdots ,1,\cdots ,0\right]}^{T}.$$

### 3.3. Imitation Learning Neural Network

**Figure 3:**
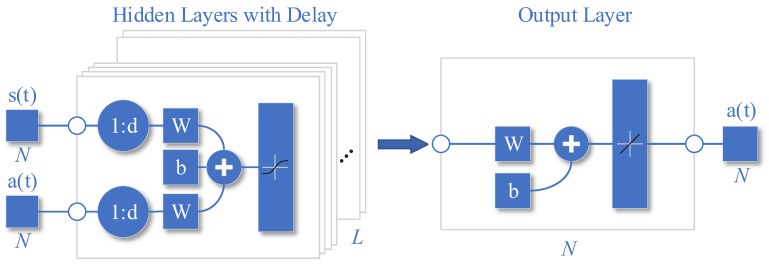
Imitation Learning Neural Network.

As shown in Figure 3, it is a schematic diagram of the network structure of the trained ILNN. The trained ILNN should predict the next maximum reward action based on the past state and action of the past d timeslots:

(12) $$a_{t}=f_{\mathrm{NARX}}\left(s_{t-d},\cdots ,s_{t-1};a_{t-d},\cdots ,\;a_{t-1}\right).$$

Here, the neural network $f_{\theta }$ can be expressed as a mapping from the input state domain S to the output action domain A, that is, $f_{\theta }:S\to A$. Considering each hidden layer as a transformation function, the neural network can be expressed as

(13) $$f_{\theta }\left(s\right)=g\circ f_{L}\circ f_{L-1}\circ \cdots f_{2}\circ f_{1}\left(s\right)$$

where *g* is the output layer transformation function, $f_{i}$ is the *i*th hidden layer, and *L* is the number of hidden layers, the hidden layer can be expressed as follows:

(14) $$f_{H}\left(s\right)=f_{L}\circ f_{L-1}\circ \cdots f_{2}\circ f_{1}\left(s\right)$$

where $f_{i}\circ f_{i-1}=f_{i}\left(f_{i-1}\left(•\right)\right)$ and $f_{1}$ to $f_{L}$ are the transformation functions of the hidden layer. The transformation function of the hidden layer and the output layer can be broken down into linear transformation and nonlinear transformation. The linear transformation is the product of the weight vector and the input vector plus the bias variable, and the nonlinear transformation is the nonlinear activation. Then $f_{i}\left(•\right)$ can be expressed as follows:

(15) $$f_{i}\left(x\right)=\sigma \left(w_{i}^{T}x\;+\;b_{i}\right)$$

Here, $w_{i}$ and $b_{i}$ are the weight vector and bias variable of the *i*th layer, respectively.

The specific process of the network structure is expressed in the following:

(16) $$\begin{array}{cc} H= & \mathrm{Tanh}\left(w_{\mathrm{In}1}s_{I}\;+\;w_{\mathrm{In}2}a_{I}\;+\;b_{1}\right)\\ a_{t}= & w_{\mathrm{out}}H\;+\;b_{2} \end{array},$$

where H is the hidden layer output matrix, $w_{\mathrm{In}1}$ is the hidden layer historical state weight parameter group, $w_{\mathrm{In}2}$ is the hidden layer historical action weight parameter group, b1 is the hidden layer offset parameter group, $w_{\mathrm{Out}}$ is the output layer weight parameter group, and $b_{2}$ is the output layer offset parameter group.

The state input of the network is the concatenation of the state vectors of the past d timeslots, represented as $s_{I}$,

(17) $$s_{I}=\left[s_{t-d},s_{t-d+1},\cdots ,s_{t-1}\right].$$

While the action input is the concatenation of the state vectors of the past d timeslots, represented as $a_{I}$,

(18) $$a_{I}=\left[a_{t-d},a_{t-d+1},\cdots ,a_{t-1}\right].$$

The activation function used is Tanh(•),

(19) $$\mathrm{Tanh}\left(x\right)=\frac{e^{x}-e^{-x}}{e^{x}+e^{-x}}=\frac{2}{1+e^{-2x}}-1.$$

due to its fully differentiable, antisymmetric, and symmetric center at the origin characteristics, making it a commonly used activation function of the hidden layer.

The network training process is formalized in

(20) $${\hat{a}}_{t}=\underset{\theta }{\arg\min}{∥\mu \left(\Pi_{\theta }\right)-\mu_{E}∥}_{2}=\underset{\theta }{\arg\min}∥E\left[\sum_{t=0}^{\infty }R\left({\hat{a}}_{t},s_{t}\right)|\Pi_{\theta }\right]{-\frac{1}{m}\sum_{i=1}^{m}\sum_{t=0}^{\infty }R\left(a_{t},s_{t}\right)∥}_{2}$$

where θ is the network parameter group, including $w_{\mathrm{In}1}$, $w_{\mathrm{In}2}$, $b_{1}$, $w_{\mathrm{Out}}$, $b_{2}$. The network parameters are adjusted to minimize the gap between the reward of the network strategy and the expert strategy, obtaining the output result ${\hat{a}}_{t}$ of imitation learning.

### 3.4. Decision Making

The output ${\hat{a}}_{t}$ of the ILNN is an N∗1 sequential array of decimal numbers. To reduce the influence of error on channel selection and make channels with similar values have similar selection probability, ${\hat{a}}_{t}$ is used as the selection probability of decision action after exponential compression and normalization, represented by

(21) $${\hat{a}}_{t}^{*}=\exp\left({\hat{a}}_{t}\right)-1,P_{\hat{a}}=\frac{{\hat{a}}_{t}^{*}-\min\left(a_{t}^{*}\right)}{{∥{\hat{a}}_{t}^{*}-\min\left({\hat{a}}_{t}^{*}\right)∥}_{1}}.$$

where $P_{\hat{a}}$ is the selection probability of the anti-jamming action $a_{t}$ obtained by channel selection according to $P_{\hat{a}}$ probability.

## 4. Simulation

This section presents the simulation results of this manuscript from four aspects: jamming settings, simulation settings, performance experiments, and comparative experiments. The jamming settings part introduces a jamming environment where jamming appears in various patterns alternately. The simulation setting introduces the definition of several important concepts in this section. The performance experiments part demonstrates the impact of different numbers of hidden layers in the neural network and sample delays on the anti-jamming performance. The comparative experiments part compares the proposed method in this manuscript with a DQN-based anti-jamming method. However, our source code is unfortunately not available due to the requirements of the organization. We will actively communicate with any researcher who is interested in this paper and send us an email.

### 4.1. Jamming Setting

In the scenario of this manuscript, the corresponding state samples are as follows. Taking *M* timeslots as a sample, each sample is divided into *k* parts with long random timeslot lengths and their jamming parameters are randomly generated. Then a jamming sample is given by:

(22) $$S=\left[s_{1},s_{2},\cdots ,\;s_{M}\right],$$

among them, there are k−1 time nodes, $1<T_{1}<T_{2}<\cdots <T_{k-1}<M$, such that

(23) $$\begin{array}{c} \left[s_{1}:s_{T_{1}}\right]=J_{1}\left(t\right)+J_{D}\left(t\right)\\ \left[s_{T_{1}+1}:s_{T_{2}}\right]=J_{2}\left(t\right)+J_{D}\left(t\right)\\ \vdots \\ \left[s_{T_{k-1}+1}:s_{M}\right]=J_{k}\left(t\right)+J_{D}\left(t\right), \end{array}$$

here, $J_{1}\left(t\right),\cdots J_{k}\left(t\right)\in \left\{J_{S}\left(t\right),J_{H}\left(t\right),J_{C}\left(t\right),J_{W}\left(t\right)\right\}$, $J_{S}\left(t\right)$ is the SJ, $J_{H}\left(t\right)$ is the NSJ, $J_{C}\left(t\right)$ is the CJ, $J_{\mathrm{CS}}\left(t\right)$ is the CSJ, and $J_{D}\left(t\right)$ is the DJ.

**Figure 4:**
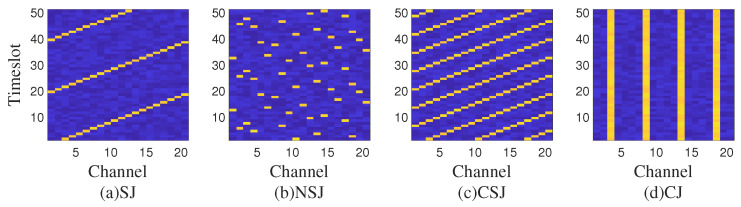
Time-frequency waterfall figure of the spectrum anti-jamming process.

In this manuscript, we consider a scenario where two different malicious jammers are present: the determined jammer and the dynamic jammer. As shown in Figure 4, they use different jamming techniques, and they jam in the following ways, respectively:

***Determined jammer*** (DJ): The jammer selects a channel for a period of time to continuously jam the channel.

***Dynamic jammer***: The jammer alternately uses Sweep Jamming (SJ), Nonlinear Sweep Jamming (NSJ), Comb Jamming (CJ), and Comb Sweep Jamming (CSJ).

Among them, for a single channel, the jamming signal is blocking jamming, that is, in the channel where the jamming signal is located, the communication cannot communicate normally. The research focus of the anti-jamming decision problem is to select a channel and transmit power in time and effectively to ensure reliable transmission of wireless communication systems by means of machine learning and neural networks.

Each jamming signal can be represented as follows.

#### 4.1.1. Sweep Jamming

(24) $$s_{S}\;=\;J_{S}\left(t;\theta_{S1},\theta_{S2},P_{S}\right)\;=\;\left[s^{*},c_{\left(\theta_{S1}\;+\;\theta_{S2}\cdot t\right)\;\mathrm{mod}\;FL}=P_{S}\right]$$

where $\theta_{S1}$ is the start channel of the sweep jamming, $\theta_{S2}$ is the channel offset of the sweep jamming, that is, the next moment is several channels to the right compared with the previous moment, $P_{S}$ is the sweep jamming power. $s^{*}={\left[0,0,\cdots ,0\right]}^{T}$, $\left[s^{*},c_{\left(\theta_{S1}\;+\;\theta_{S2}\cdot t\right)\;\mathrm{mod}\;FL}=P_{S}\right]$ means that on the basis of $s^{*}={\left[0,0,\cdots ,0\right]}^{T}$, the $\left(\theta_{S1}\;+\;\theta_{S2}\cdot t\right)\;\mathrm{mod}\;FL$th term $c_{\left(\theta_{S1}\;+\;\theta_{S2}\cdot t\right)\;\mathrm{mod}\;TL}$ is equal to $P_{S}$. In general, in order to traverse all channels, $\theta_{S2}$ and FL should have no common factors other than 1.

#### 4.1.2. Nonlinear Sweep Jamming

(25) $$s_{H}\;=\;J_{H}\left(t;\mathrm{map},T_{H},P_{H}\right)\;=\;\left[s^{*},c_{\mathrm{map}\left(t\;\mathrm{mod}\;T_{H}\right)}=P_{H}\right]$$

where map(•) is the nonlinear sweep pattern, $T_{H}$ is the nonlinear sweep period, and $P_{S}$ is the nonlinear sweep jamming power.

#### 4.1.3. Comb Jamming

(26) $$s_{C}\;=\;J_{C}\left(t;\theta_{C1},\theta_{C2},P_{C}\right)\;=\;\left[s^{*},c_{\theta_{C1}\;\mathrm{mod}\;\theta_{C2}:\theta_{C2}:FL}=P_{C}\right]$$

where $\theta_{C1}$ is the comb-jamming starting channel, $\theta_{C2}$ is the comb-jamming channel interval, and $P_{C}$ is the comb-jamming power. $\theta_{C1}\;\mathrm{mod}\;\theta_{C2}:\theta_{C2}:FL$ represents an array starting from $\theta_{C1}\;\mathrm{mod}\;\theta_{C2}$, separated by $\theta_{C2}$, and not exceeding FL at most. For example, $\theta_{C1}=7$, $\theta_{C2}=4$, $\theta_{C1}\;\mathrm{mod}\;\theta_{C2}:\theta_{C2}:FL=3,7,11,15,19$. Then the comb jamming signal $J_{C}\left(t\right)\;=\;\left[s^{*},c_{3,7,11,15,19}=P_{C}\right]$.

#### 4.1.4. Comb Sweep Jamming

(27) $$s_{W}\;=\;J_{W}\left(t;\theta_{W1},\theta_{W2},\theta_{W3},P_{W}\right)=\left[s^{*},c_{\left(\theta_{W1}\;+\;\theta_{W2}\cdot t\right)\;\mathrm{mod}\;\theta_{W3}:\theta_{W3}:FL}=P_{W}\right]$$

where $\theta_{W1}$ is the initial frequency of comb sweep jamming, $\theta_{W2}$ is the channel shift of comb sweep jamming, $\theta_{W1}$ is the channel interval of comb sweep jamming, and $P_{W}$ is the power of comb sweep jamming.

#### 4.1.5. Determined Jamming

(28) $$s_{D}\;=\;J_{D}\left(t;\theta_{D1},P_{D}\right)=\left[s^{*},c_{\theta_{D1}}\;=\;P_{D}\right]$$

where $\theta_{D1}$ is the jamming channel of fixed-frequency jamming, and $P_{D}$ is the fixed-frequency jamming power.

### 4.2. Simulation Setting

The software and hardware information of the performance experiment is shown in Table 2. We used MATLAB (2021a) to generate the data, Python (3.8) to perform machine learning, and the data were stored in .mat files. The machine learning library we used was TensorFlow1.14.

The simulation parameters of the performance experiment are shown in Table 3. Simulation parameters mean that this manuscript considers a multi-channel communication scenario with 20 channels, each with a channel bandwidth of 2 MHz, and takes 1000 timeslot as a communication unit, in which the aforementioned jamming pattern will alternate randomly.

The simulation experiments focus on the collision rate as the main performance metric, specifically examining the performance variations of the proposed approach when the jamming pattern switches, the jamming-to-noise ratio (JNR) changes, and the proportion of jamming signals changes. The key concepts are defined as follows:

**Collision rate:** The ratio of the number of collisions between the communication signal and the jamming signal in the same timeslot in the same channel to the total number of timeslots.

**Jamming-to-noise ratio (JNR):** The decibel ratio of the jamming signal strength in a specific time slot and channel to the intensity of the background Gaussian noise signal in that timeslot. Generally, a lower JNR indicates less noticeable jamming, making it more challenging for the ILNN to make decisions and resulting in a higher collision rate.

**Proportion of jamming signals:** The ratio of the cumulative number of channels occupied by the jamming signal over a certain number of timeslots to the product of the number of timeslots and the number of channels of interest. Generally, a higher proportion of jamming signals indicates fewer available channels and greater difficulty in resisting jamming, resulting in a higher collision rate.

In the performance experiments, the influence of the number of hidden layers and the number of sample delays on network performance is studied. The key concepts are:

**Hidden:** The number of hidden layers of the ILNN. Generally, a higher number of hidden layers indicates a stronger expressive power of the neural network, smaller fitting errors, but also longer training and execution times.

**Delay:** The number of past timeslot samples used by the ILNN for decision-making. Generally, a larger number of delays provides more information for the ILNN to make decisions, leading to more informed decisions. However, it also makes it more difficult to converge when the jamming pattern changes, as it is more influenced by the previous pattern.

### 4.3. Performance Experiment

**Figure 5:**
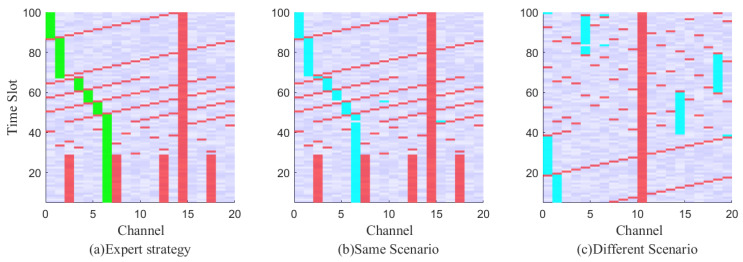
Time-frequency waterfall figure of the spectrum anti-jamming process. The red block represents the jamming signal, the lavender is the background noise, the green block is the communication trajectory selected according to the expert strategy, and the blue block is the user communication trajectory selected after imitation learning.

As depicted in Figure 5, the spectrum anti-jamming process is graphically displayed. Figure 5a illustrates a schematic diagram of channel selection based on expert strategy. The green block represents the channel selected according to the expert strategy, the red block represents jamming, and the blue-purple block represents the noise background. Figure 5b shows the channel selection method obtained through real-time decision using the imitation strategy under the same scenario as Figure 5a, and the blue block represents the channel selected according to real-time decision. In Figure 5c, the channel selection method obtained through the imitation strategy after a real-time decision is presented in a different scenario from Figure 4a. The proposed method efficiently solves the decision problem in dynamic jamming scenarios.

Below is a study on the impact of different parameters of ILNN on anti-jamming performance, with a focus on the number of hidden layers and sample delay in ILNN. Simulations were conducted to observe the collision rate as a function of JNR and the proportion of jamming signals under different ILNN parameters. The network training utilized scaled conjugate gradient which requires less memory and has faster training speed, with a total of 1000 iterations.

**Figure 6:**
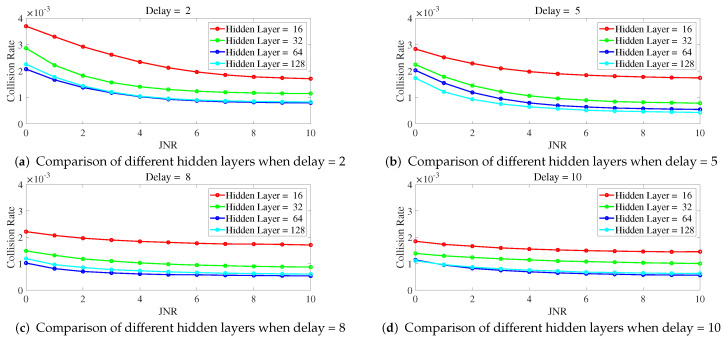
The delay is fixed, and the collision rate cha nges curve with JNR under different hidden layers.

Figure 6 illustrates the variation of collision rate as JNR changes for different numbers of hidden layers when a delay is fixed. From Figure 6, it can be observed that with a fixed delay, a higher number of hidden layers leads to a lower collision rate. In particular, the curve for ILNN with 16 hidden layers shows significantly higher collision rates compared to other curves, indicating inadequate expressive power with 16 hidden layers. On the other hand, the curves for ILNN with 64 and 128 hidden layers exhibit similar trends, and even at a delay of 8, as shown in Figure 6c, the ILNN with 64 hidden layers demonstrates a lower collision rate. This suggests that the ILNN with 128 hidden layers has excessive expressive power under this condition and, due to limited training iterations, cannot be adequately trained, resulting in similar or even lower anti-jamming performance compared to the ILNN with 64 hidden layers.

**Figure 7:**
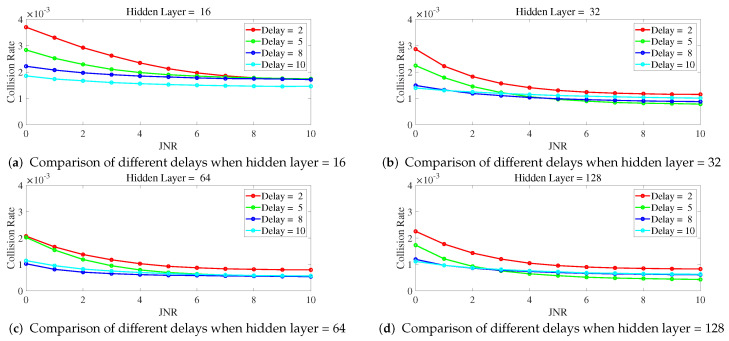
The hidden layers is fixed, and the collision rate changes curve with JNR under different delay.

Figure 7 shows the variation of collision rate as JNR changes for different delays when the number of hidden layers is fixed. From Figure 7, it can be observed that with a fixed number of hidden layers, a higher delay leads to a lower collision rate. Specifically, the curves for ILNN with a delay of 2 show noticeably higher collision rates compared to the other curves, indicating that the ILNN with a delay of 2 lacks sufficient historical samples as input for making decisions, leading to inadequate anti-jamming capability. The curves for delay = 8 and delay = 10 are similar, with performance lower than that of delay = 8. This is because the limited training iterations prevent them from being adequately trained.

**Figure 8:**
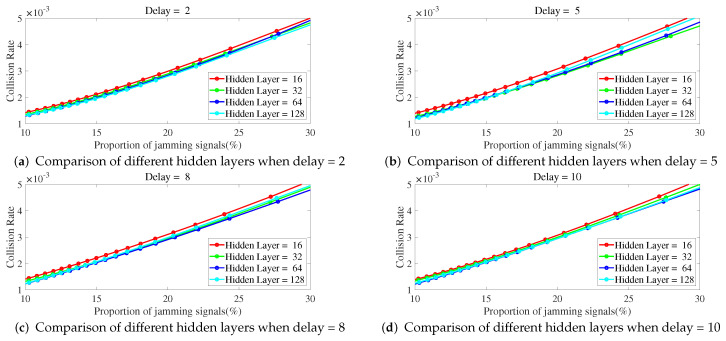
The delay is fixed, and the collision rate changes curve with the proportion of jamming signals under different hidden layers.

**Figure 9:**
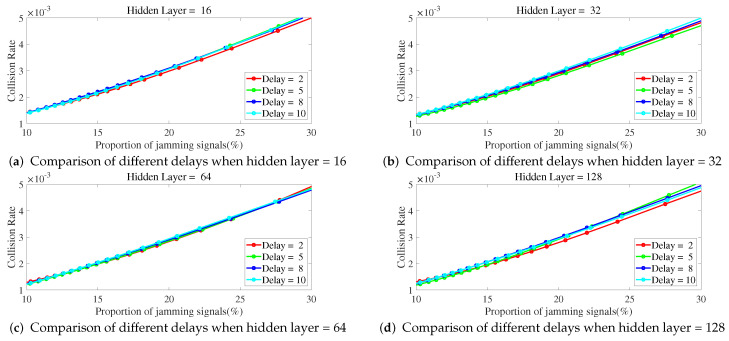
The hidden layers are fixed, and the collision rate changes curve with the proportion of jamming signals under different delays.

As shown in Figure 8 and Figure 9, the collision rate changes with the proportion of jamming signals. As the proportion of jamming signals increases, the collision rate also increases. As shown in Figure 8, when the delay is fixed, the number of hidden layers is less and the jamming collision rate is higher. The ILNN with 16 hidden layers has the highest jamming collision rate.

As shown in Figure 9, when the number of hidden layers is fixed, the change of the delay number has little impact on the collision rate when the proportion of jamming signals increases. Therefore, when the proportion of jamming signals increases, it is not feasible to improve the performance of ILNN by only increasing the delay number and participating in more samples of the past time slots without increasing the number of hidden layers of the network.

In summary, increasing the number of hidden layers and delay values can enhance the anti-jamming performance of ILNN when the JNR or proportion of jamming signals increases. However, excessive hidden layers and high delay values do not significantly improve the system’s anti-jamming performance, while also requiring longer training time. Furthermore, higher delay values make the anti-jamming decision more susceptible to the influence of the previous jamming pattern. In the simulation scenario of this manuscript, an ILNN with 128 hidden layers does not show better anti-jamming performance compared to an ILNN with 64 hidden layers. ILNNs with a delay of 8 or 10 do not exhibit better anti-jamming performance compared to an ILNN with a delay of 5. Therefore, in this simulated scenario, an ILNN with 64 hidden layers and a delay of 5 can ensure both anti-jamming performance and a smaller network size. Hence, the comparative experiment in Section 4.4 selects an ILNN with 64 hidden layers and a delay of 5.

### 4.4. Comparative Experiments

This subsection presents a comparative analysis between IL-based anti-jamming methods, RL-based anti-jamming methods, and DQN-based anti-jamming methods. It examines the performance curves and the time required for re-stabilization of the three methods when there is a sudden switch in the jamming pattern. Moreover, it investigates the variations in collision rate changes for the three methods as the JNR varies. Additionally, it examines the variations in collision rates for the three methods as the proportion of jamming signals fluctuates.

**Figure 10:**
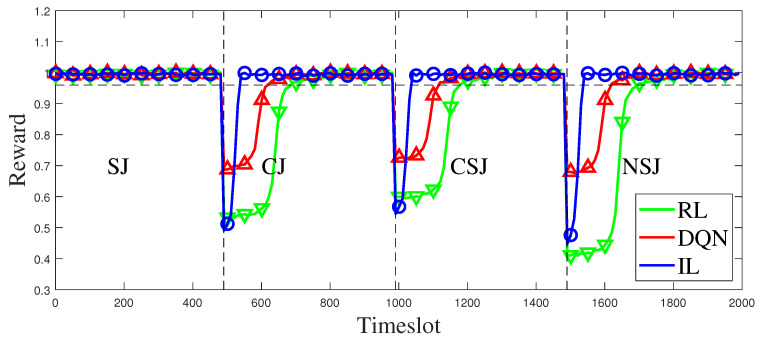
Comparison of anti-jamming performance among RL, DQN and IL when jamming pattern is switched.

A comparison is made among the proposed methods, RL and DQN. Figure 10 illustrates the performance transformation curves of IL, converged RL, and converged DQN when faced with sudden switching of jamming patterns. Based on the simulation results, it can be observed that the RL-based and the DQN-based anti-jamming decision methods take a long time to reconverge when the jamming pattern switches, whereas the proposed method can return to a stable state faster despite a sharp performance drop. Apart from the three switching processes depicted in Figure 10, additional details on the duration of the transitions can be found in Table 4, Table 5 and Table 6. In comparison to RL and DQN, which learn and decide simultaneously as the states change, IL utilizes imitation and generalization from expert strategy that encompass certain jamming-switching processes. Consequently, when the jamming pattern switches, IL demonstrates reduced “decision inertia” toward previous jamming patterns, facilitating more prompt and robust decision-making. Thus, the IL-based approach proposed in this study enables the timely and effective selection of appropriate channels during jamming-type switches, ensuring the reliability and stability of communication systems.

**Figure 11:**
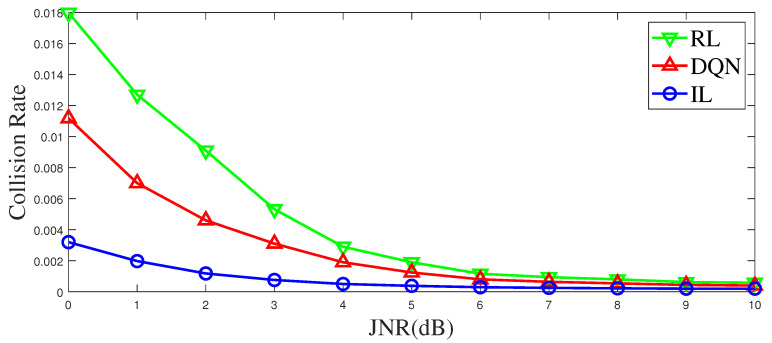
The collision rate varies with the JNR of jamming signals.

Background noise and channel fading can influence the perception of jamming, which in turn affects the anti-jamming communication. For classical Q-learning, the state space is limited, and the state value is discrete. Generally, a threshold is used to make a decision before learning, but this process causes information loss, affecting learning and decision-making. In this study, the noisy state is directly input to DQN and IL for anti-jamming decisions. For RL, noise affects their sensing of the environment, that is, a large number of misjudgments are generated when it makes a decision on the environment state according to the threshold, which affects the anti-jamming performance of the system. The results are presented in Figure 11. When considering channel fading and noise, IL has a lower jamming probability than RL and DQN. The IL-based method generalizes the expert strategy from a global perspective, while the RL-based method and the DQN-based methods focus on the change of the current state. So, noise and fading in the IL-based method have an impact on the decision with more overall statistical characteristics, while in the RL-based method and the DQN-based method, noise and fading have an impact on the decision with more individual random characteristics. Therefore, the IL-based method is less affected by noise and fading.

**Figure 12:**
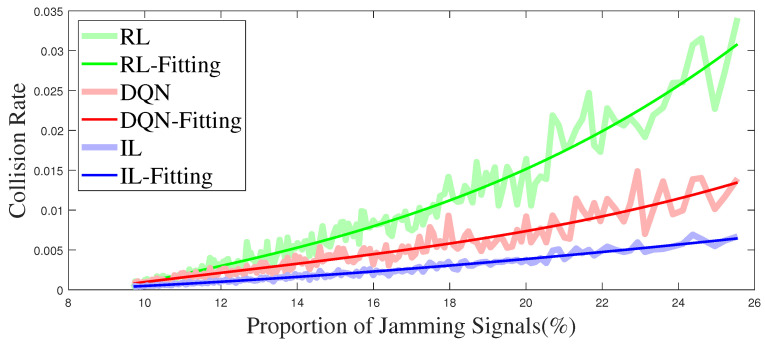
The collision rate varies with the proportion of jamming signals.

Figure 12 illustrates the relationship between the collision rate and the proportion of jamming signals. The light thick line in the figure is the direct result of the simulation, and the thin line is the curve obtained by fitting. It can be seen from Figure 12 that the collision rate of the IL anti-jamming method is about 30% lower than that of the DQN-based method and 80% lower than that of the RL-based method under the same proportion of jamming signals. The increase in the proportion of jamming signals means that more channels are jammed, and the RL-based and the DQN-based methods have fewer decision choices in the learning process, making the decision more difficult. However, the training method based on IL has different parameters and expert strategy under different jamming patterns, the influence of an increasing proportion of jamming signals is minimal.

The aforementioned experiments demonstrate that the IL-based anti-jamming method exhibits faster re-stabilization during sudden changes in jamming patterns when compared to the RL-based method and the DQN-based anti-jamming method. Moreover, the IL-based approach shows reduced susceptibility to background noise and channel fading, enabling prompt and effective multi-channel decisions in response to variations in the environment. Additionally, when faced with increasing proportions of jamming signals, the IL-based method proves more efficient in avoiding collisions compared to RL and DQN.

## 5. Conclusions

The proposed anti-jamming method based on imitation learning generates expert trajectories from historical samples, which are then imitated using ILNN. Users can obtain anti-jamming strategies from the trained ILNN for anti-jamming decision-making. This method enables timely and effective multi-channel decisions in response to changes in the jamming environment. Compared to the DQN-based anti-jamming method, this method exhibits faster convergence when faced with sudden switches in jamming patterns. It is less affected by background noise and channel fading, resulting in a lower collision rate when the proportion of jamming signals increases.

However, this method has limited generalization capability and limited ability to handle jamming patterns beyond those contained in the training samples. Further research and improvement can be conducted by incorporating deep reinforcement learning. The combination of imitation learning, which learns from historical information, and deep reinforcement learning, which adapts to real-time changes in the current environment, can be explored. Additionally, as the number of hidden layers in ILNN increases, its expressive power is enhanced but requires more training time. Moreover, there is a threat of adversarial samples to ILNN. Adversaries can significantly impact the anti-jamming decision-making of ILNN at minimal cost. Targeted optimization can be applied to ILNN by leveraging adversarial training techniques in adversarial machine learning.

In future work, we will consider combining imitation learning with deep reinforcement learning to make more robust intelligent anti-jamming communication decisions, aiming at the problem of poor adaptability when facing new scenarios. Aiming at the problem that imitation learning requires a high number of historical samples, it was considered to combine it with the generative adversarial network, so that the system could train an excellent enough ILNN with limited historical samples.

The advantage of imitation learning lies in obtaining a posteriori optimal trajectory through the analysis of historical samples, and obtaining a better experience for anti-jamming by imitating the posteriori optimal trajectory. Its disadvantage lies in requiring more historical samples for the analysis of posteriori optimal trajectory. Deep reinforcement learning, on the other hand, focuses on solving the problem of the moment, can quickly learn from a new environment, and can perform as it learns. Our initial idea is to trust the results of deep reinforcement learning more when there are fewer historical samples or historical experience has a low anti-jamming reference value to the current scene, and trust the results of imitation learning more when there are enough state experience samples collected for imitation learning. Through such a combination, the system can obtain both the flexibility of deep reinforcement learning and the high accuracy of imitation learning. In the process of combining IL and DQN, we need to increase the storage space complexity and the computational complexity of each timeslot, but we can obtain an anti-jamming communication method with higher accuracy and fewer timeslots. The combined system will converge faster and the convergence value will be higher.

## Figures and Tables

**Table 1:** Summary of Related Research.

Reference	Year	Research Area	Technical Scheme	Method	Weakness
[10]	2020	LTE & LoRa	Automated Laboratory Measurement	Automatic Control Method	Historical experience is underutilized
[11]	2020	IoT	Automated Measurement		
[12]	2023	5G	FDRL	FL	Performance is sacrificed for security
[13]	2022	Secrecy-driven FL	FL		
[14]	2022	FANET	AFRL		
[15]	2021	Beamforming	Meta Learning	Meta-L	High computing power requirements
[16]	2022	Image Classification	MGML		
[17]	2020	Jamming Recognition	Meta Learning		
[18]	2021	Wireless sensor networks	JMAA	RL	Curse of dimensionality; Unable to cope with continuous-state Spaces
[19]	2022	UAV Anti-Jamming Communication	CMRL		
[20]	2022	Deceiving-based anti-jamming methods	Reinforcement Learning		
[21]	2018	Frequency Selection of HF Communication	Deep Reinforcement Learning	DRL	Low sampling efficiency; Unsteadiness
[22]	2023	Autonomous Vehicle Networks	Deep Reinforcement Learning		
[23]	2022	UAV Swarm Network	UCB-DQN		

**LTE**: The long term evolution of Universal Mobile Telecommunications System; **LoRa:** Long Range Radio; **FDRL:** Federated Deep Reinforcement Learning; **AFRL:** Adaptive Federated Reinforcement Learning; **MGML:** Momentum Group Meta-Learning; **JMAA:** Joint Multi-agent Anti-jamming Algorithm; **CMRL:** Collaborative Multiagent Reinforcement Learning; **UCB-DQN:** Upper Confidence Bound Deep Q Network; **FL:** Federated learning; **Mate-L:** Mate learning; **RL:** Reinforcement Learning; **DRL:** Deep Reinforcement Learning.

**Table 2:** Hardware and Software.

Parameters	Value
CPU	i7-12700K
GPU	RTX-3060Ti
Data Generation Environment	MATLAB (2021a)
Machine Learning Environment	Python (3.8)
Data Storage Format	.mat
Algorithms Library	TensorFlow 1.14

**Table 3:** Simulation Parameters.

Parameters	Value
Layers	16, 32, 64, 128
Delay	2, 5, 8, 10
Length of Timeslot	1 ms
Channel Bandwidth	2 MHz
Number of Channels	20
Number of Timeslots for one communication unit	1000

**Table 4:** Duration of restabilization after jamming pattern switch of RL.

	After	CJ	SJ	CSJ	NSJ
Before					
CJ		0	225.49	219.41	246.92
SJ		239.46	0	233.14	243.27
CSJ		256.71	202.22	0	250.06
NSJ		251.79	206.98	230.69	0

**Table 5:** Duration of restabilization after jamming pattern switch of DQN.

	After	CJ	SJ	CSJ	NSJ
Before					
CJ		0	120.06	115.81	145.27
SJ		125.85	0	124.08	152.43
CSJ		143.91	96.34	0	142.46
NSJ		138.91	91.97	135.01	0

**Table 6:** Duration of restabilization after jamming pattern switch of IL.

	After	CJ	SJ	CSJ	NSJ
Before					
CJ		0	63.75	58.25	39.58
SJ		47.97	0	57.96	46.51
CSJ		45.63	31.15	0	45.99
NSJ		57.71	34.19	62.40	0

## Data Availability

The data presented in this study are not available due to privacy.
